# Comparative Assessment of Phytochemical Compounds and Antioxidant Properties of Kernel Oil from Eight Sour Cherry (*Prunus cerasus* L.) Cultivars

**DOI:** 10.3390/molecules27030696

**Published:** 2022-01-21

**Authors:** Małgorzata Stryjecka, Monika Michalak, Jacek Cymerman, Anna Kiełtyka-Dadasiewicz

**Affiliations:** 1 Institute of Agricultural Sciences, State School of Higher Education Chełm, 22-100 Chełm, Poland; mstryjecka@pwszchelm.edu.pl (M.S.); jcymerman@pwszchelm.edu.pl (J.C.); 2 Department of Dermatology, Cosmetology and Aesthetic Surgery, Collegium Medicum, Jan Kochanowski University, 25-369 Kielce, Poland; 3 Department of Plant Production Technology and Commodity Science, University of Life Sciences, 20-950 Lublin, Poland

**Keywords:** *Prunus cerasus*, sour cherry oil, fatty acids, phytochemicals, antioxidants

## Abstract

New plant oils as a potential natural source of nutraceutical compounds are still being sought. The main components of eight cultivars (‘Koral’, ‘Lucyna’, ‘Montmorency’, ‘Naumburger’, ‘Wanda’, ‘Wigor’, ‘Wołyńska’, and ‘Wróble’) of sour cherry (*Prunus cerasus* L.) grown in Poland, including crude fat, protein, and oil content, were evaluated. The extracted oils were analysed for chemical and biological activity. The oils had an average peroxide value of 1.49 mEq O_2_/kg, acid value of 1.20 mg KOH/g, a saponification value of 184 mg of KOH/g, and iodine value of 120 g I_2_/100 g of oil. The sour cherry oil contained linoleic (39.1–46.2%) and oleic (25.4–41.0%) acids as the major components with smaller concentrations of α-eleostearic acid (8.00–15.62%), palmitic acid (5.45–7.41%), and stearic acid (2.49–3.17%). The content of sterols and squalene varied significantly in all the studied cultivars and ranged between 336–973 mg/100 g and 66–102 mg/100 g of oil. The contents of total tocochromanols, polyphenols, and carotenoids were 119–164, 19.6–29.5, and 0.56–1.61 mg/100 g oil, respectively. The cultivar providing the highest amounts of oil and characterised by the highest content of PUFA (including linoleic acid), plant sterols, α-and β-tocopherol, as well as the highest total polyphenol and total carotenoids content was been found to be ‘Naumburger’. The antioxidant capacity of sour cherry kernel oils, measured using the DPPH^•^ and ABTS^•+^ methods, ranged from 57.7 to 63.5 and from 38.2 to 43.2 mg trolox/100 g oil, respectively. The results of the present study provide important information about potential possibilities of application of *Prunus cerasus* kernel oils in cosmetic products and pharmaceuticals offering health benefits.

## 1. Introduction

Plant oils are an important part of human diet, but also an essential ingredient of many cosmetic and pharmaceutical products. The nutritional and technological value of oils is determined mainly by the composition of fatty acids, but also by the content of substances, such as polyphenols, tocopherols, and carotenoids. Plant oils, including oils from fruit seeds, rich in biological compounds, exert beneficial effects on the human body (improved wellbeing and health status, reduced risk of diseases, e.g., cardiovascular diseases, neurological disorders, certain cancers, obesity, or type 2 diabetes) and significantly affect the proper appearance and function of the skin [1,2,3]. The above properties of plant-derived oils rich in health-enhancing compounds are associated, inter alia, with their ability of scavenging free radicals [4]. The pharmaceutical and cosmetic industries are looking for innovative sources of functional bio-oils rich in bioactive compounds. The presence of phytochemicals and nutraceuticals in sour cherry fruit justifies further use of kernels from *Prunus cerasus* L. species to produce oils for food, pharmaceutical, and cosmetic applications [5,6].

The sour cherry (*Prunus cerasus* L.) belonging to the Rosaceae family, is a species cultivated in many regions of the world due to its fruits that are of great importance in the food industry for the production of juices, jams, and frozen food [6]. Poland is a leading producer of cherry fruits for consumption (a total of over 270,000 tons in 2017 and 2018) and is ranked fourth in the world after Russia, Turkey, and Ukraine [7]. The cherry fruit processing generates significant amounts of wastes (seeds) that can be used for oil production. For this purpose, however, the data on oil efficiency and quality are needed. Preliminary data of other authors inspire to obtain oil also from cherry cultivars growing in Poland. Górnaś et al. [8] have described the differences in the yield and quality of oil from cherry seeds of different cultivars. In the present study, the cultivars grown in the largest quantities for industrial purposes in Poland, i.e., ‘Koral’, ‘Lucyna’, ‘Montmorency’, ‘Naumburger’, ‘Wanda’, ‘Wigor’, ‘Wołyńska’, and ‘Sparrows’, were examined and described. The inedible residue of cherry fruit processing is a pit containing the oil, which is a rich source of polyunsaturated and monounsaturated fatty acids, as well as lipophilic compounds, such as carotenoids, tocochromanols, phytosterols, and squalene, showing considerable beneficial health effects [2,5,8]. Literature data indicate that the content of active ingredients in sour cherry kernel oils depends on cultivars and agro-climatic conditions [2,5]. The studies in this area are rather limited and concern sour cherry cultivars from Hungary [9], Romania [10], Greece [11], Italy [2], Turkey [5,12,13], Iran [6], and the USA [14]. Considering the growing interest in sour cherry-based products, it is essential to increase our knowledge of the chemical composition and biological activity of kernel oil from various *P. cerasus* L. cultivars. Therefore, the aim of the present study was to examine the main components (crude fat, protein, and oil) of kernels from eight cultivars of sour cherry (*P. cerasus* L.) grown in Poland. Moreover, the physicochemical properties of the sour cherry kernel oil, including acid value, peroxide value, saponification value, iodine value, fatty acid composition, content of sterols, and squalene, were determined. In addition, tocopherols and tocotrienols, total carotenoid, and total polyphenol content, as well as antioxidant activity (DPPH^•^ and ABTS^•+^) were comprehensively investigated.

## 2. Results and Discussion

### 2.1. Sour Cherry Kernel Properties

The content of crude fat and crude protein in kernel of eight sour cherry cultivars was analysed. The inter-cultivar differences were slight (Table 1). The mean crude fat and crude protein content of sour cherry kernel were 41.3 and 31.3%, respectively. The results obtained for the content of crude protein were generally consistent with those reported by Kazempour-Samak et al. [6] 29.3% and Lazos [11] 25.3% [11]. *P. cerasus* kernels were found to be rich in oil (31.6%, on average); however, the differences for individual cultivars were noticeable. According to another study, the sour cherry kernel contained 36.1 [13], 31.89 [6], 26.0 [11], 22.5 [10], and 17.0% [12] of oil.

As shown in Table 1, the highest amount of oil was found in ‘Naumburger’ (35.4%), while the lowest content was detected in ‘Koral’ (24.6%). According to the results of another study, the oil content in different sour cherry cultivars grown in Poland, including ‘Haritonovskaya’, ‘Latvijas Zemais’, ‘Shokoladnica’, and ‘Zentenes’, was 37.1, 36.2, 36.6, and 35.2%, respectively [8].

### 2.2. Characteristics of Sour Cherry Kernel Oil

Some of the chemical and physical properties of the oils obtained from *P. cerasus* kernels are shown in Table 2. The mean peroxide value was 1.49 mEq O_2_/kg oil, which was below the acceptable level for cold pressed or virgin oils according to the Codex Alimentarius Commission [15]. ‘Koral’ was found to have the lowest peroxide value amongst all the tested samples, i.e., 1.37 mEq O_2_/kg oil. The peroxide value of sour cherry kernel oil from Romania, was reported to be 1.6 mEq O_2_/kg oil [10], while from Iran—0.99 mEq O_2_/kg oil [6]. Many factors are responsible for the high peroxide value, including oxygen availability and temperature among others [16].

The iodine value indicates the degree of unsaturation of fatty acids and exhibits stability of the oil against oxidation [6]. The iodine values of sour cherry oil of eight cultivars varied from 118 to 123 g of I2/100 g of oil, which is consistent with the findings reported by Lazos [11] (116 g of I2/100 g of oil) and Popa et al. [10] (122.5 g of I2/100 g of oil). Otherwise, the values determined were lower than those reported by Kazempour-Samak et al. [6] (130.99 g of I2/100 g of oil).

The results revealed that the mean saponification value of the oil sample was 184 mg KOH/g oil, which is in line with the findings reported by Popa et al. [10] (183 mg KOH/g oil). However, the highest saponification number of the ‘Montmorency’ (191 mg KOH/g oil) was lower than the values reported previously for sour cherry oil, i.e., 193 mg KOH/g oil [17], 194 mg KOH/g oil [6], and 198 mg KOH/g oil [11]. The free fatty acids in a sample were measured using the acid value assay. In the present study, the mean acid value of eight tested sour cherry oils was 1.20 mg KOH/g oil, which was lower than the values reported in another study—1.36 mg KOH/g oil [6] and 1.45 mg KOH/g oil [17]. Increased saponification and acid values indicate progressive oxidative processes, which adversely affect the quality of oils and is usually caused by storage changes and their duration. Thus, the comparison of these values with the literature data may be associated with the risk of error; nevertheless, it shows the rate of oxidation of the studied oils from various cultivars stored under identical conditions.

### 2.3. Fatty Acid Composition of Sour Cherry Kernel Oil

Table 3 shows the contents of fatty acids detected in oils obtained from kernels of various *P. cerasus* cultivars. Some inter-cultivar differences in the content of dominant fatty acids, including linoleic (39.1–46.2%) and oleic (25.4–41.0%) acid, as well as α -eleostearic (8.00–15.62%), palmitic (5.45–7.41%), and stearic (2.49–3.17%) acid were observed.

The above findings are consistent with the results reported by other authors [5,8,10,11,13]. Expectedly, the content of linoleic acid (42.5%) was higher than that of oleic acid (36.7%), which is consistent with some previously reported data [6,8]. An exception was ‘Montmorency’, whose content of oleic acid was higher (41.0%), as compared to the content of linoleic acid (39.1%), which is in agreement with the results of other authors, i.e., 42.9 and 38.2 [10], 45.8 and 41.8 [13], 46.0 and 41.6 [11], 63.9 and 27.0 [5], and 46.3% and 41.5% [12], respectively.

The above results demonstrate that the content of dominant fatty acids depends on the cherry cultivar from which the oil was obtained. Moreover, α-eleostearic acid was found to be in third place, following oleic and linoleic acids and its content depended on the cultivar (8.00–15.62%). The study findings are similar to the results reported by other authors, showing that the content of α-eleostearic acid was 9.34 [6] or 7.43–15.76% [8]. In the present study, the content of palmitic acid ranged from 5.45 to 7.41% and the stearic acid value ranged from 2.49 to 3.17%, which is similar to the results reported previously where the content of palmitic acid was 5.3 [5], 6.4 [12], 6.54 [6], 6.9 [13], 5.06–7.38 [8], 8.60 [11], and 11% [10] followed by the content of stearic acid, being 1.2 [12], 1.5 [5], 2.03 [6], 2.22–3.45 [8], 2.6 [13], 2.86 [11], and 6.4% [10]. The minor content (below 2%) of fatty acids in the oils evaluated in the present study was noted for arachidic acid, palmitoleic acid, α-linolenic acid, and gondoic acid. 

The differences in the content of individual fatty acids in oils from different sour cherry cultivars are associated with the differences in total saturated, total unsaturated, monounsaturated, and polyunsaturated fatty acids (Table 4). The content of total unsaturated fatty acids (UFA) (monounsaturated and polyunsaturated fatty acid) was higher than that of saturated fatty acids (SFA). All oil samples had high amounts of total unsaturated fatty acids, mainly linoleic acid and oleic acid. Moreover, the polyunsaturated fatty acid level (47.9–62.2%) was higher than that of monounsaturated fatty acids (26.2–41.8%) in all the samples studied. Similar results reported by Górnaś et al. have demonstrated higher percentages of PUFA (44.0–62.3%) compared to MUFA (26.0–46.1%) in oils obtained from kernels of various sour cherry cultivars [6]. The results of the present study are consistent with the findings reported by Kazempour-Samak et al., according to which the content of PUFA and MUFA was 52.66% and 36.14%, respectively [6]. Different results were reported by Uluata and Özdemir; in their study the content of PUFA (42.4%) was found to be lower than that of MUFA (49.1%) [13]. The ratios of Ʃ UFA/ Ʃ SFA and Ʃ PUFA/ (Ʃ SFA+ Ʃ MUFA) in the oil from kernels of different *P. cerasus* cultivars were within the range of 8.11–9.59 and 0.92–1.65, respectively, which is comparable to the findings reported by Górnaś et al., i.e., 7.5–9.6 and 0.8–1.7, respectively [8].

The composition of fatty acids is of particular importance for the nutritional value of oil. SFA and MUFA can be synthesised in the human body while PUFA cannot be due to the lack of enzymatic systems capable of introducing double bonds in the n-6 and n-3 positions of the carbon chain and have to be supplied daily with food. The ratio of acids from the n-6 to n-3 family (4–5:1, without exceeding the value of 10:1 for adults) in the diet is important, because an excess of n-6 fatty acids inhibits the metabolism of n-3 fatty acids, which may lead to a physiological imbalance of hormone-like substances synthesised from them called eicosanoids and docosanoids (including prostaglandins (PGs), thromboxanes (TXs), prostacyclin (PGI2), leukotrienes (LTs), resolvins (RVD), and other lipid mediators) [18]. The results obtained in the present paper show that sour cherry kernel oil is characterized by a favourable quantitative ratio of n-6/n-3 fatty acids.

**Figure 1:**
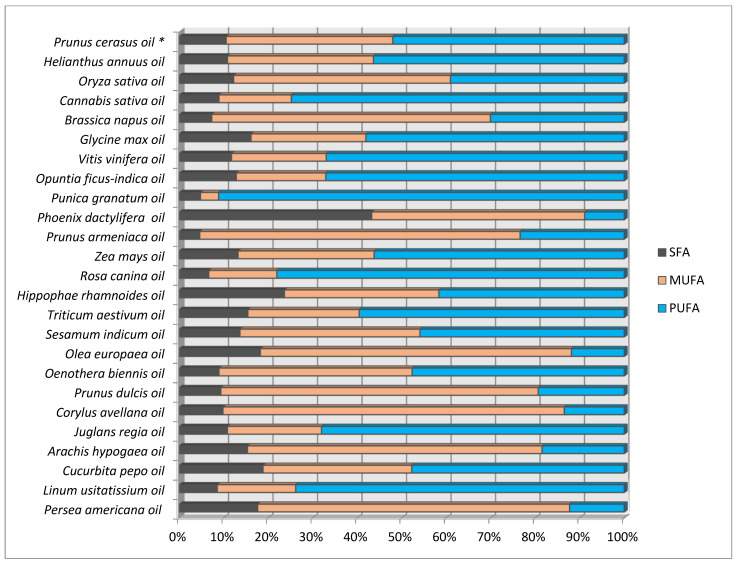
Saturated fatty acids (SFA), monounsaturated fatty acids (MUFA), and polyunsaturated fatty acids (PUFA) in sour cherry oils (mean for eight cultivars) in comparison with selected conventional plant oils. Source: * data obtained from our own research; all other data from literature [21,22,23,24,25,26].

The vegetable oils obtained from seeds or fruits of plants traditionally considered to be oily raw materials (rapeseed, sunflower, olive, and soybean oil), but also oils from atypical vegetable raw materials (pumpkin oil, grape, hemp, evening primrose, and others) can be used both as ingredients of nutritional (food) supplements, nutricosmetics and cosmetics (Figure 1). SFA determine the stability of the oil, while PUFA are responsible for health benefits [19,20]. The sum of SFA (mean 10.5), MUFA (mean 37.4), and PUFA (mean 52.0%) in the tested cherry oils was found to be similar to the commonly consumed oils, such as sunflower, corn, pumpkin seed, or wheat germ oils.

Sour cherry kernel oil, with low content of saturated fatty acids and the high content of unsaturated fatty acids, is highly favourable in human nutrition and health. It is known that unsaturated fatty acids are of importance to the human body and skin health [19]. Monounsaturated oleic acid with a number of potential health effects (including improvement of immune system function, maintenance of normal blood cholesterol levels, and impact on the modulation of inflammatory markers) is a possible ingredient of functional foods [27]. Polyunsaturated linoleic acid, an omega-6 essential fatty acid, shows beneficial effects on lipid profile, glucose metabolism, and cardiovascular disease risk reduction [28]. Oleic and linoleic acids are the only unsaturated fatty acids detected in the stratum corneum [29]. Linoleic acid, a component of ceramide 1, plays an important role in the cohesiveness of the intracellular cement and proper functioning of the skin barrier limiting the transepidermal water loss (TEWL), thus ensuring adequate hydration and protecting against external factors [19].

### 2.4. Sterol Composition and Squalene Content of Sour Cherry Kernel Oil

Phytosterols, steroid compounds present in plants, cannot be synthesized by humans. They exhibit the ability to reduce LDL-cholesterol concentrations in humans, which reduces the risk of cardiovascular diseases. Moreover, sterols are believed to have anti-inflammatory, antibacterial, antiulcer, antioxidant, and anticancer activities [30]. Squalene, a natural triterpene widely distributed in plant oils, which can easily move through the cellular and subcellular membrane, exerts antibacterial, antifungal, and antioxidant effects [31]. Squalene, as the main component of skin surface polyunsaturated lipids, protects the skin cells against free radical oxidative damage and has moisturizing as well as emollient properties [32].

Besides other components of the unsaponifiable fraction, such as phospholipids, tocopherols, tocotrienols, and carotenoids, sterols and squalene give the oils a high nutritional value. Their percentages in oils depend on the type of raw material, its cultivars and climate conditions [33].

The total amount of sterols in the oils of eight cultivars ranged from 336 to 973 mg/100 g oil (Table 5). β-sitosterol was found in the largest amount. The content of this compound varied significantly depending on the cultivar. These results are consistent with those reported by Lazos [11], Górnaś et al. [8], Korlesky et al. [17], and Kazempour-Samak et al. [6]. Other major sterol compounds were ∆5-avenasterol, 24-methylene-cycloartanol, cholesterol, and campesterol, in lower amounts—gramisterol, Δ7-stigmasterol, and citrostadienol. The content of squalene in sour cherry kernel oil was considerable. The highest amount of squalene was observed in ‘Montmorency’ (102 mg/100 g oil) while the lowest one in ‘Koral’ (66 mg/100 g oil). The above amounts are comparable to the amounts of squalene in other cultivars of sour cherries grown in Poland, which ranged between 65.8 and 102.8 mg/100 g oil [8].

### 2.5. Phytochemical Content and Antioxidant Potential of Sour Cherry Kernel Oil

Tocochromanols are the important lipophilic compounds with antioxidant activity in sour cherry oil. The total concentration of tocochromanols ranges between 119 and 164 mg/100 g (Table 6). The vitamin capacity of tocopherols is related to the content of α-tocopherol (vitamin E), as the most biologically active. Vitamin E inhibits the production of new free radicals, protect against lipid peroxidation, is involved in various physiological and biochemical functions of the body, reduces the risk of cardiovascular diseases, cancer, or age-related macular degeneration, and plays an important role in maintaining skin health [19,34]. The range and average of α-T in kernel oils recovered from various sour cherry cultivars were 9.2–35.7 and 18.0 mg/100 g. According to Uluata and Özdemir [13], the content of α-T was 74.7 mg/kg; according to Matthäus and Özcan [5]—4.7 mg/kg. Some study results show that γ-tocopherol is a better negative risk factor for certain types of cancer and myocardial infarction than α-tocopherol [35]. The results obtained in this study demonstrate that the amounts of γ-T in the study oils were much higher, as compared to α-T. The differences in γ-T content in the sour cherry oils were substantial depending on the cultivar and ranged from 85.8 mg/100 g (‘Naumburger’) to 132.5 mg/100 g (‘Lucyna’). γ-T was also the main tocochromanol present in other cultivars of sour cherries grown in Poland; its levels ranged between 89.1 and 133.3 mg/100 g oil [8]. According to other authors [5,13], the content of γ-T was lower, i.e., 579.9 mg/kg oil [13], and 197.2 mg/kg oil [5].

Polyphenols constitute an important group of compounds in sour cherry oil, which are characterised by multidirectional biological activity. They exhibit anti-inflammatory, antibacterial, antifungal, antiviral, antiallergic, as well as anticancer properties. Besides carotenoids, polyphenols act as antioxidants protecting the human cells, tissues, and the entire body against the destructive effects of free radicals. They play an extremely important role in skin protection against environmental factors [19]. The average value of polyphenols in the sour cherry kernel oils was 22.8 µg/g oil (expressed as GAE) (Table 6). A comparable content of total polyphenols (18.5 μg GAE/g oil) in sour cherry kernel oils was reported previously [13]. The findings of the present study concerning the content of carotenoids (0.84 mg/100 g oil, on average) are consistent with the findings of other authors [8,12].

The free radical scavenging capacity of the studied oils, determined using the common antioxidant methods of DPPH^•^ and ABTS^•+^, ranged from 57.7–63.5 to 38.2–43.2 mg trolox/100 g oil, respectively. The results concerning the oil from cultivars of sour cherries grown in Poland were comparable with the value in the oil samples harvested in Turkey (57.4 and 38.7 mg trolox/100 g oil, respectively) reported by Uluata and Özdemir [13].

**Figure 2:**
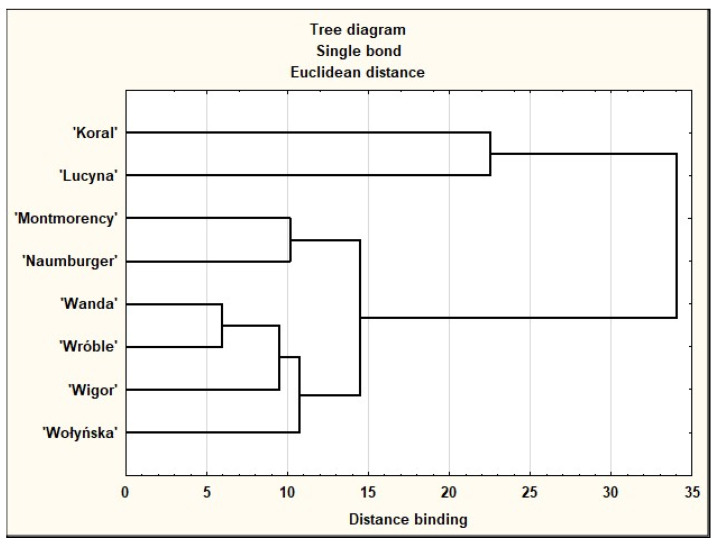
Dendrogram of eight sour cherry cultivars based on numerical data for bioactive compounds with antioxidative properties in the studied oils, including derivatives of tocopherols (α, β, γ, δ) and tocotrienols (α, γ), total tocochromanol, total carotenoid, and total polyphenol content, as well as the DPPH^•^ and ABTS^•+^ radical scavenging capacities.

Since it is difficult to compare the oils studied as to their antioxidant properties based on Table 6, the data were subjected to cluster analysis (Figure 2). Based on the dendrogram, the group of cultivars characterised by high similarity can be distinguished, i.e., ‘Wanda’, ‘Wróble’, ‘Wigor’, and ‘Wołyńska’ as well as ‘Montmorency’ and ‘Naumburger’. ‘Koral’ and ‘Lucyna’ were distinctly different from other cultivars. Moreover, the similarity between them was relatively low.

## 3. Materials and Methods

### 3.1. Plant Material 

The plant material consisted of the kernels of eight cultivars of sour cherry (*P. cerasus* L.): ‘Koral’, ‘Lucyna’, ‘Montmorency’, ‘Naumburger’, ‘Wanda’, ‘Wigor’, ‘Wołyńska’, and ‘Wróble’. Sour cherry fruits were harvested in August 2017 and 2018 from 6- and 7-year-old trees grown in an organic orchard in south-eastern Poland, near Chełm (51°7′ N, 23°28′ E). All results are reported as the mean of the two years of study, due to insignificant differences for the research seasons.

### 3.2. Sample Preparation

Immediately after harvesting, the kernels were hand-separated from the fruit, washed with tap water, and oven dried at 40 °C (Memmert GmbH & Co. KG, Germany, Schwabach ) for 24 h. After removal of the seed coating, seed kernel samples (50 g of each cultivar) were crushed in a commercial blender (MICROTRON^®^ MB 550, Kinematica AG, Malters, Switzerland). The crushed material, packed in a paper thimble, was placed in a Soxhlet extractor connected to a condenser and a 500 mL round-bottom flask. The extraction was performed with diethyl ether (250 mL) in a water bath for 6 h. Then the solvent was removed under reduced pressure using a rotary evaporator (Rotavapor^®^ R-100, Buchi Labortechnik AG, Flawil, Switzerland).

### 3.3. Analysis of the Main Components of Kernels

The content of crude fat was determined using the AOAC Official Method [36]. Crude fat was dried in Na_2_SO_2_ (anhydrous sodium sulphate); subsequently, the remaining solvent was evaporated in an evaporator and the pure oil obtained was determined qualitatively.

The protein content was determined in the defatted kernels by the Kjeldahl method according to the International Organization for Standardization (ISO) [37].

### 3.4. Analysis of the Chemical Properties and Oxidative Stability of Oils

The selected chemical properties of the oils were determined, i.e., saponification, peroxide, acid, and iodine values. The measurements were performed with an AT 1000 automatic titrator. The following were determined: the saponification value according to ISO 3657:2013 [38] and titrated with 0.5 mol/L HCl. The peroxide value was determined according to the ISO 3960:2007 [39] standard method. The samples were titrated with a 0.002 N solution of sodium thiosulfate. The free fatty acid (FFA) content determined using the ISO 660:2009 [40] standard protocol. A 1 g sample of oil was dissolved in 20 mL of a mixed dichloromethane/ethanol (1/1, *v*/*v*) solution and titrated with 0.1 M potassium hydroxide solution. The acid value by multiplying the FFA value by a factor of 1.99. The iodine value determined using the iodine monochloride (Wijs solution), as described in the ISO 3961:2009 [41] method. The samples were titrated with 0.1 N sodium thiosulfate.

### 3.5. Analysis of Fatty Acid Composition

Fatty acid profiles were measured by gas chromatography according to ISO standards [42,43]. The oil samples (100 mL each) were converted to their fatty acid methyl esters (FAMEs). The FAME samples were analysed in a gas chromatograph (Shimadzu GC-2010 PLUS) equipped with a flame ionisation detector. A highly polar BPX 70 capillary column (60 m × 0.25 mm, 25 μm) was used for separation. The column was programmed in a temperature range between 140 and 210 °C, the dosing temperature was 210 °C, and the detector temperature was set at 250 °C. The carrier gas was 6.0 helium, with a constant flow rate of 2 mL/min.

### 3.6. Determination of Tocochromanols, Total Carotenoids, and Polyphenols

Tocopherol and tocotrienol homologues were determined by HPLC (HP 1100 with a UV detector) according to PN-EN 12822:2014-08 [44]. The analysis was carried out in the reversed-phase mode. The oil sample was dissolved in methanol and applied to the top of a Supelcosil LC-18 column (25 cm × 4.6 mm, 5 μm). The separation was carried out at 30 °C. The mobile phase was methanol/water (97:3, *v*/*v*) and the flow rate was 1 mL/min. Individual α, γ, and δ isomers were determined at 292 nm. Total tocochromanols were calculated by summing up the contents of all tocopherols and tocotrienols.

Total carotenoids were determined spectrophotometrically; 0.2 g of oil sample was diluted with n-hexane in 5 mL volumetric flasks and the absorbance was measured at 450 nm with a UV-2600i spectrophotometer (Shimadzu, Kyoto, Japan).

The total carotenoid concentration in oil samples was spectrophotometrically quantitated using the molar extinction coefficient for all-trans-β-carotene (ε = 139,049) and converted equations of the Beer–Lambert law and molar concentration.

c = (A/ε) × l m = c × MW × V(1)

where c—concentration (mol/L); A—absorbance; ε—molar extinction coefficient (L/mol × cm); l—path-length (cm); m—mass of total carotenoids in the sample (g); MW—molecular weight (g/mol); and V—volume of solution (L).

Determination of the total polyphenol content (TPC) was based on the Folin–Ciocalteu total phenolic assay [45], using gallic acid as a reference standard. Absorbance of the samples was measured with a UV–Vis spectrophotometer (UV-2600, Shimadzu, Kyoto, Japan) at 725 nm. The results were expressed as gallic acid equivalent (µg GAE/g oil).

### 3.7. Total Antioxidant Capacity

The extracts containing the polar fraction of oils were prepared according to Szydłowska-Czerniak et al. [46]. A 10 mL volume of methanol and water (70:30 *v*/*v*) was added to 2 g of oil and extraction was carried out for 1 h without access to light. The samples were then frozen at −20 °C for another hour until the layers were separated. The extract was separated from the oil by quantitative transfer to glass flasks and stored in a freezer (−20 °C) until analysis.

The antioxidant capacity values of methanolic extracts were determined using DPPH^•^ (1,1-diphenyl-2-picrylhydrazyl) assay according to the method described by Singh et al. [47]; 0.1 mL extract and 2 mL methanolic DPPH solution were mixed. The mixture was vigorously shaken and incubated at room temperature for 30 min. The absorbance was recorded at 517 nm by a spectrophotometer (UV-2600i, Schimadzu, Kyoto, Japan).

The ABTS^•+^ antioxidant activity was determined according to Re et al. [48]. Firstly, the ABTS radical was prepared as 7 mM ABTS water solutions and 2.45 mM potassium persulfate (1:0.5 ratio, *v*:*v*), which were mixed and diluted with ethanol until the absorbance (at 734 nm) was 0.70. Subsequently, 1 mL of ABTS radical was added into 20 μL of sample. The absorbance was read at 734 nm. The results were given as trolox equivalent.

### 3.8. Statistical Analysis

Analysis of all parameters in the oil samples was performed in triplicate. Statistica 9.0 StatSoft was used to analyse the data. One-way ANOVA with the HSD Tukey test was used to evaluate the significant inter-mean differences at *p* < 0.05. A dendrogram was obtained by hierarchical cluster analysis, considering the numerical data for all parameters described in Table 6, using the Euclidean distances between individual data points.

## 4. Conclusions

Chemical characteristics and antioxidant properties of oils from eight cultivars of sour cherries grown in Poland were analysed in this study. The results revealed that sour cherry kernels contained appreciable amounts of oil rich in several classes of pharmacological active compounds with known therapeutic uses. As a high oleic–linoleic oil, it can be extremely important for human health due to its superior stability and nutritional value. Moreover, the presence of bioactive compounds, such as tocopherols, carotenoids, and polyphenols in sour cherry kernel oils, demonstrates the possibility of their further applications in cosmetics and pharmaceuticals offering health benefits.

## Figures and Tables

**Table 1:** The main components of sour cherry kernels of eight cultivars.

Cultivar	Components		
	Crude Fat (%)	Crude Protein (%)	Oil (%)
‘Koral’	41.3 ^C^	31.6 ^AB^	24.6 ^F^
‘Lucyna’	40.9 ^D^	31.4 ^BC^	31.1 ^D^
‘Montmorency’	41.8 ^A^	31.7 ^A^	32.5 ^C^
‘Naumburger’	41.6 ^B^	30.9 ^E^	35.4 ^A^
‘Wanda’	41.3 ^C^	31.5 ^B^	33.5 ^B^
‘Wigor’	41.1 ^C^	31.1 ^D^	32.8 ^BC^
‘Wołyńska’	41.2 ^C^	31.3 ^CD^	29.1 ^E^
‘Wróble’	40.9 ^D^	31.1 ^DE^	33.5 ^B^
Mean	41.3	31.3	31.6

Values marked with the same superscript letter do not differ significantly at *p* < 0.05.

**Table 2:** Quality parameters of oils from kernel of eight sour cherry cultivars.

Cultivar	Acid Value (mg KOH/g)	Saponification Value (mg KOH/g)	Peroxide Value (mEq O_2_/kg)	Iodine Value (g I_2_/100 g)
‘Koral’	1.00 ^D^	182 ^DE^	1.37 ^D^	118 ^D^
‘Lucyna’	1.11 ^C^	186 ^BC^	1.45 ^BCD^	118 ^D^
‘Montmorency’	1.36 ^A^	191 ^A^	1.53 ^ABC^	119 ^CD^
‘Naumburger’	1.32 ^A^	185 ^BC^	1.63 ^A^	123 ^A^
‘Wanda’	1.13 ^C^	181 ^DE^	1.58 ^AB^	120 ^BC^
‘Wigor’	1.21 ^B^	183 ^CD^	1.48 ^BCD^	121 ^B^
‘Wołyńska’	1.20 ^B^	187 ^B^	1.43 ^CD^	121 ^B^
‘Wróble’	1.12 ^C^	180 ^E^	1.47 ^BCD^	121 ^B^
Mean	1.20	184	1.49	120

Values marked with the same superscript letter do not differ significantly at *p* < 0.05.

**Table 3:** Fatty acid composition in oils from different sour cherry cultivars.

Cultivar	Fatty Acid Content (%)								
	C 16:0	C 16:1	C 18:0	C 18:1	C18:2	α-C18:3	α-ESA C 18:3	C 20:0	C 20:1
‘Koral’	5.95 ^F^	0.23 ^D^	3.17 ^A^	38.4 ^C^	42.2 ^D^	0.20 ^B^	8.18 ^B^	1.12 ^F^	0.47 ^C^
‘Lucyna’	6.43 ^D^	0.33 ^AB^	2.78 ^D^	37.5 ^D^	41.3 ^F^	0.12 ^CD^	8.13 ^B^	1.19 ^E^	0.60 ^A^
‘Montmorency’	6.10 ^E^	0.29 ^BC^	2.91 ^C^	41.0 ^A^	39.1 ^G^	0.21 ^B^	8.58 ^B^	1.23 ^D^	0.50 ^C^
‘Naumburger’	7.41 ^A^	0.32 ^AB^	2.96 ^B^	25.4 ^F^	46.2 ^A^	0.43 ^A^	15.62 ^A^	1.34 ^A^	0.43 ^D^
‘Wanda’	5.45 ^H^	0.35 ^A^	2.70 ^E^	39.2 ^B^	42.0 ^E^	0.11 ^DE^	8.45 ^B^	1.29 ^BC^	0.56 ^B^
‘Wigor’	5.82 ^G^	0.30 ^BC^	2.61 ^F^	39.1 ^B^	42.3 ^C^	0.09 ^E^	8.00 ^B^	1.31 ^AB^	0.49 ^C^
‘Wołyńska’	6.49 ^C^	0.26 ^CD^	2.81 ^D^	35.2 ^E^	44.6 ^B^	0.21 ^B^	8.69 ^B^	1.26 ^CD^	0.47 ^C^
‘Wróble’	7.32 ^B^	0.25 ^D^	2.49 ^G^	37.5 ^D^	42.4 ^C^	0.14 ^C^	8.50 ^B^	1.19 ^E^	0.34 ^E^
Mean	6.37	0.29	2.81	36.7	42.5	0.19	9.27	1.24	0.48

C 16:0, palmitic acid; C 16:1, palmitoleic acid; C 18:0, stearic acid; C 18:1, oleic acid; C 18:2, linoleic acid; α-C18:3, α-linolenic acid; α-ESA C 18:3-α-eleostearic acid; C 20:0-arachidic acid; C 20:1-gondoic acid. Values marked with the same superscript letter do not differ significantly at *p* < 0.05.

**Table 4:** Sum of SFA, MUFA, and PUFA (%), and fatty acid ratios of sour cherry kernel oils.

Cultivar	Ʃ SFA	Ʃ MUFA	Ʃ PUFA	Fatty Acids Ratio Ʃ UFA/Ʃ SFA	Fatty Acids Ratio Ʃ PUFA/ (Ʃ SFA+ ƩMUFA)	PUFA Ratio n-6/n-3
‘Koral’	10.3 ^C^	39.1 ^C^	50.6 ^CD^	8.75 ^C^	1.02 ^C^	5.03 ^A^
‘Lucyna’	11.4 ^C^	38.8 ^C^	49.5 ^E^	8.46 ^D^	1.01 ^C^	5.00 ^A^
‘Montmorency’	10.2 ^C^	41.8 ^A^	47.9 ^F^	8.76 ^C^	0.92 ^D^	4.46 ^B^
‘Naumburger’	11.7 ^A^	26.1 ^F^	62.2 ^A^	7.54 ^F^	1.65 ^A^	2.87 ^C^
‘Wanda’	9.4 ^D^	40.1 ^B^	50.5 ^D^	9.59 ^A^	1.02 ^C^	4.91 ^AB^
‘Wigor’	9.7 ^D^	39.9 ^B^	50.4 ^D^	9.27 ^B^	1.02 ^C^	5.22 ^A^
‘Wołyńska’	10.6 ^C^	36.0 ^E^	53.5 ^B^	8.47 ^D^	1.15 ^B^	5.01 ^A^
‘Wróble’	11.0 ^B^	38.0 ^D^	51.0 ^C^	8.11 ^E^	1.04 ^C^	4.91 ^AB^
Mean	10.5	37.4	52.0	8.62	1.10	4.68

Ʃ MUFA, sum of monounsaturated fatty acids; Ʃ PUFA, sum of polyunsaturated fatty acids; Ʃ SFA, sum of saturated fatty acids; Ʃ UFA, sum of unsaturated fatty acids. Values marked with the same superscript letter do not differ significantly at *p* < 0.05.

**Table 5:** Sterol and squalene content (mg/100 g oil) in sour cherry kernel oils.

Cultivar	Sterols										Squalene
	Campesterol	β-Sitosterol	Δ5-Avenasterol	24-Methylene-Cycloartanol	Cholesterol	Gramisterol	Δ7-Stigmasterol	Δ7-Avenasterol	Citrostadienol	Total	
‘Koral’	9.7 ^C^	260 ^F^	20.5 ^C^	24.7 ^B^	9.3 ^C^	3.6 ^D^	2.17 ^CD^	3.07 ^C^	3.13 ^B^	336 ^F^	66 ^F^
‘Lucyna’	7.7 ^DE^	284 ^E^	15.7 ^EF^	22.6 ^C^	12.3 ^B^	3.3 ^E^	N.D.	2.33 ^D^	N.D.	348 ^E^	69 ^E^
‘Montmorency’	22.7 ^B^	732 ^B^	74.2 ^B^	29.1 ^A^	2.7 ^D^	11.4 ^B^	10.2 ^B^	5.57 ^B^	5.03 ^A^	893 ^B^	102 ^A^
‘Naumburger’	39.4 ^A^	790 ^A^	76.7 ^A^	29.7 ^A^	2.6 ^D^	12.4 ^A^	10.8 ^A^	6.03 ^A^	5.40 ^A^	973 ^A^	78 ^C^
‘Wanda’	8.2 ^D^	318 ^C^	15.4 ^EF^	21.9 ^CD^	14.3 ^A^	6.1 ^C^	2.33 ^C^	1.77 ^E^	2.27 ^C^	390 ^C^	75 ^D^
‘Wigor’	7.2 ^EF^	253 ^G^	16.2 ^D^	20.8 ^DE^	12.7 ^B^	2.7 ^F^	1.77 ^D^	1.47 ^EF^	2.57 ^C^	318 ^G^	82 ^B^
‘Wołyńska’	8.0 ^D^	285 ^E^	15.7 ^EF^	21.6 ^CD^	12.2 ^B^	3.3 ^E^	1.63 ^DE^	1.30 ^F^	N.D.	348 ^E^	82 ^B^
‘Wróble’	7.1 ^F^	291 ^D^	15.0 ^F^	20.3 ^E^	12.8 ^B^	2.9 ^F^	1.17 ^E^	1.53 ^EF^	2.53 ^C^	354 ^D^	80 ^BC^
Mean	13.8	401	31.2	23.9	9.9	5.69	4.30	2.88	3.49	495	79.3

N.D. not detected. Values marked with the same superscript letter do not differ significantly at *p* < 0.05.

**Table 6:** Phytochemical compounds with antioxidant effects in sour cherry kernel oils.

Cultivar	Tocochromanols (mg/100 g Oil)							TC (mg/100 g)	TP (µg GAE/g)	DPPH (mg Trolox/100 g)	ABTS^•+^ (mg Trolox/100 g)
	α-T	β-T	γ-T	δ-T	α-T3	γ-T3	Total				
‘Koral’	9.2 ^G^	0.40 ^F^	123.7 ^B^	10.5 ^G^	0.87 ^D^	0.3 ^B^	145 ^B^	0.56 ^D^	19.6 ^G^	57.9 ^EF^	38.2 ^G^
‘Lucyna’	10.2 ^F^	0.50 ^E^	132.5 ^A^	18.6 ^A^	1.67 ^B^	0.4 ^A^	164 ^A^	0.59 ^D^	19.6 ^G^	58.2 ^E^	39.4 ^E^
‘Montmorency’	29.1 ^B^	1.17 ^B^	88.6 ^F^	12.4 ^D^	0.60 ^E^	0.3 ^B^	132 ^D^	1.06 ^B^	28.5 ^B^	62.7 ^B^	40.4 ^D^
‘Naumburger’	35.7 ^A^	2.33 ^A^	85.8 ^G^	11.7 ^E^	2.17 ^A^	0.2 ^C^	138 ^C^	1.61 ^A^	29.5 ^A^	61.2 ^C^	43.2 ^A^
‘Wanda’	14.6 ^D^	0.58 ^DE^	98.2 ^C^	9.7 ^H^	0.75 ^D^	0.3 ^B^	124 ^F^	0.77 ^C^	22.7 ^C^	63.1 ^AB^	39.1 ^EF^
‘Wigor’	18.5 ^C^	0.68 ^C^	91.2 ^E^	14.2 ^C^	1.13 ^C^	0.2 ^C^	126 ^E^	0.73 ^C^	21.6 ^D^	63.5 ^A^	38.7 ^FG^
‘Wołyńska’	12.7 ^E^	0.60 ^CD^	88.6 ^F^	15.7 ^B^	0.87 ^D^	0.2 ^C^	119 ^G^	0.63 ^D^	20.2 ^F^	57.7 ^F^	42.5 ^B^
‘Wróble’	14.1 ^D^	0.60 ^CD^	96.8 ^D^	11.3 ^F^	0.53 ^E^	0.2 ^C^	124 ^F^	0.81 ^C^	20.7 ^E^	58.7 ^D^	41.8 ^C^
Mean	18.0	0.86	100.7	13.0	1.07	0.3	134	0.84	22.8	60.4	40.4

ABTS^•+^, 2,2′-azinobis-(3-ethylbenzothiazoline-6-sulfonic acid); DPPH^•^, 2,2′-diphenyl- 1-picrylhydrazyl; GAE, gallic acid equivalent; α-T, α -tocopherol; β-T, β-tocopherol; γ-T, γ-tocopherol; δ-T, δ-tocopherol; α-T3, α-tocotrienol; γ-T3, γ-tocotrienol; TC, total carotenoids; TP, total polyphenols. Values marked with the same superscript letter do not differ significantly at *p* < 0.05.

## Data Availability

The data presented in this study are available on request from the corresponding authors.

## Sample Availability

Samples of oils are available from the authors.
